# Neuroblastoma and DIPG Organoid Coculture System for Personalized Assessment of Novel Anticancer Immunotherapies

**DOI:** 10.3390/jpm11090869

**Published:** 2021-08-30

**Authors:** Waleed M. Kholosy, Marc Derieppe, Femke van den Ham, Kim Ober, Yan Su, Lars Custers, Linda Schild, Lieke M. J. van Zogchel, Lianne M. Wellens, Hendrikus R. Ariese, Celina L. Szanto, Judith Wienke, Miranda P. Dierselhuis, Dannis van Vuurden, Emmy M. Dolman, Jan J. Molenaar

**Affiliations:** Princess Máxima Center for Paediatric Oncology, Heidelberglaan 25, 3584 CS Utrecht, The Netherlands; m.p.p.derieppe-3@prinsesmaximacentrum.nl (M.D.); f.vandenham-2@prinsesmaximacentrum.nl (F.v.d.H.); K.Ober@prinsesmaximacentrum.nl (K.O.); Y.Su@prinsesmaximacentrum.nl (Y.S.); l.m.c.custers@prinsesmaximacentrum.nl (L.C.); G.G.Schild@prinsesmaximacentrum.nl (L.S.); l.vanzogchel@sanquin.nl (L.M.J.v.Z.); l.m.wellens@prinsesmaximacentrum.nl (L.M.W.); H.C.R.Ariese@prinsesmaximacentrum.nl (H.R.A.); C.L.Szanto-2@prinsesmaximacentrum.nl (C.L.S.); j.wienke-4@prinsesmaximacentrum.nl (J.W.); M.P.Dierselhuis@prinsesmaximacentrum.nl (M.P.D.); D.G.vanVuurden@prinsesmaximacentrum.nl (D.v.V.); M.E.M.Dolman-2@prinsesmaximacentrum.nl (E.M.D.); j.j.molenaar@prinsesmaximacentrum.nl (J.J.M.)

**Keywords:** neuroblastoma, diffuse intrinsic pontine glioma (DIPG), paediatric cancer organoids, coculture system, cancer immunotherapy

## Abstract

Cancer immunotherapy has transformed the landscape of adult cancer treatment and holds a great promise to treat paediatric malignancies. However, in vitro test coculture systems to evaluate the efficacy of immunotherapies on representative paediatric tumour models are lacking. Here, we describe a detailed procedure for the establishment of an ex vivo test coculture system of paediatric tumour organoids and immune cells that enables assessment of different immunotherapy approaches in paediatric tumour organoids. We provide a step-by-step protocol for an efficient generation of patient-derived diffuse intrinsic pontine glioma (DIPG) and neuroblastoma organoids stably expressing eGFP-ffLuc transgenes using defined serum-free medium. In contrast to the chromium-release assay, the new platform allows for visualization, monitoring and robust quantification of tumour organoid cell cytotoxicity using a non-radioactive assay in real-time. To evaluate the utility of this system for drug testing in the paediatric immuno-oncology field, we tested our in vitro assay using a clinically used immunotherapy strategy for children with high-risk neuroblastoma, dinutuximab (anti-GD2 monoclonal antibody), on GD2 proficient and deficient patient-derived neuroblastoma organoids. We demonstrated the feasibility and sensitivity of our ex vivo coculture system using human immune cells and paediatric tumour organoids as ex vivo tumour models. Our study provides a novel platform for personalized testing of potential anticancer immunotherapies for aggressive paediatric cancers such as neuroblastoma and DIPG.

## 1. Introduction

Recent developments in cancer immunotherapy hold great promise to treat aggressive paediatric malignancies such as high-risk neuroblastoma and diffuse intrinsic pontine glioma (DIPG) [1,2]. However, suitable preclinical models to predict the efficacy of immunotherapeutics are largely lacking. Therefore, the establishment of robust ex vivo coculture systems of paediatric tumour cells and immune cells is of great importance to accelerate the pace of translating novel immunotherapeutics into clinical applications.

Recent advances in organoid technology have paved the way to generate more physiologically relevant and personalized preclinical tumour models. These are well suited for use in coculture systems to study drug effects dependent on interactions between tumour cells and other cells in the tumour microenvironment such as immunotherapeutics. Patient-derived organoids isolated from paediatric tumour tissues offer the advantage of recapitulating the morphological and functional tumour complexity and can be used as superior ex vivo tumour models for compound testing [3,4,5,6].

Immunotherapeutic modalities are diverse, including targeted antibodies, small molecules, immune checkpoint modulators, adoptive cell therapies, oncolytic viruses, and cancer vaccines. Most of these approaches aim to either unleash or potentiate the cytotoxic effector immune cell function to recognize and destroy tumours. Current in vitro methods to evaluate the efficacy of immunotherapies aim at either direct measurement of tumour cell death or surrogate markers involved in the cytotoxic effector cell activity such as cytokine release. In particular, the chromium-release assay has been developed more than 40 years ago and is still considered the gold standard method to measure T- or NK-cell mediated tumour cell cytotoxicity [7,8]. Due to its biohazard and spontaneous isotope leakage issues, alternative biochemical and cell-based methods were developed that measure ATP, proteases, Lactate dehydrogenase (LDH) or other enzymes released by dying cells, thereby eliminating the use of radioactive materials. These methods are mostly laborious, less sensitive, and not suitable to differentiate between cytotoxic effects on tumour cells and immune cells in a multicellular coculture setting. Furthermore, these tests are mostly incompatible with high-throughput screening (HTS) workflows [9,10,11]. Recently, bioluminescence-based assays have emerged as alternative methods to selectively measure tumour cell viability in vitro and in vivo [12,13]. In these methods, tumour cell lines are transduced to express endogenous luciferase that catalyses the emission of a bioluminescent signal that can be detected and measured using luminometers. Despite success in tumour cell lines, efficient transduction of difficult-to-manipulate primary tumour cells such as patient-derived neuroblastoma and DIPG tumour organoids in serum-free conditions has thus far been challenging.

To overcome the above limitations, we developed a bioluminescence-based ex vivo coculture system to enable the assessment of different immunotherapy approaches in paediatric tumour organoids. We have adopted an efficient method to generate stably transduced patient-derived organoids for paediatric cancer using serum-free culture conditions. In this study, patient-derived neuroblastoma organoids and DIPG organoids were successfully transduced with a lentiviral vector carrying enhanced green fluorescent protein—firefly luciferase (eGFP-ffLuc) fusion reporter genes. In contrast to the chromium-release assay, the new platform allows visualization, monitoring, and robust quantification of immune cell-mediated cytotoxic effects on tumour cells using a non-radioactive assay in real-time. To evaluate the utility of this system for drug testing in the paediatric immuno-oncology field, we tested and validated the effect of a clinically approved anti-GD2 immunotherapy on eGFP-ffLuc expressing patient-derived neuroblastoma organoids. Our study provides a proof-of-concept to demonstrate the feasibility and value of generating cocultures of patient-derived tumour organoids and immune cells as novel model systems for the more reliable and robust testing of potential immunotherapy-based treatment approaches for paediatric cancer.

## 2. Materials and Equipment

### 2.1. Reagents

DMEM—GLUTAMAX (Life Technologies, Merelbeke, Belgium, cat. no. 21885)

Ham’s F-12 Nutrient Mixture (F-12) (Life Technologies, Merelbeke, Belgium, cat. no. 31765-027)

Penicillin–streptomycin (Life Technologies, Merelbeke, Belgium, cat. no. 15140-122)

N-2 supplement, 100X=× (Life Technologies, Merelbeke, Belgium, cat. no. 17502048)

PBS (Life Technologies, Merelbeke, Belgium, cat. no. 17502048)

B27 supplement Minus Vitamin A, 50X (Life Technologies, Merelbeke, Belgium, cat. no. 12587010)

Recombinant Human Fibroblast Growth Factor-basic (FGF)-2 (PeproTech, Rocky Hill, NJ, USA, cat. no. 100-18B)

Recombinant Human Epidermal Growth Factor (EGF) (PeproTech, Rocky Hill, NJ, USA, cat. no. AF-100-15)

Recombinant Human Insulin-Like Growth Factor-I Protein (IGF-1) (R&D Systems, MN, USA, cat. no. 291-G1-200)

Recombinant Human Platelet-Derived Growth Factor-AA (PDGF-AA) (PeproTech, Rocky Hill, NJ, USA, cat. no. 100-13A)

Recombinant Human Platelet-Derived Growth Factor-BB (PDGF-BB) (PeproTech, Rocky Hill, NJ, USA, cat. no. 100-14B)

FACS buffer (BD Pharmingen, Franklin Lakes, NJ, USA, cat. no. 554657)

Accutase solution (Sigma Aldrich, St. Louis, MO, USA, cat. no. A6964)

Advanced DMEM/F-12 (Thermo Fisher, Waltham, MA, USA, cat. no. 12634010)

Neurobasal-A Medium (Thermo Fisher, Waltham, MA, USA, cat. no. 10888022)

HEPES Buffer Solution (1M) (Thermo Fisher, Waltham, MA, USA, cat. no. 15630056)

MEM non-Essential Amino Acids Solution, 100X (Thermo Fisher, cat. no. 11140035)

Glutamax™ Supplement (Thermo Fisher, Waltham, MA, USA, cat. no. 35050038)

Sodium Pyruvate 100 mM, 100× (Thermo Fisher, Waltham, MA, USA, cat. no.11360070)

Primocin 500 mg (InvivoGen, San Diego, USA, cat. no. ant-pm-1)

Heparin solution (Stem Cell Technologies, Vancouver, BC, Canada, cat. no. 7980)

MEM non-Essential Amino Acids Solution, 100× (GIBCO, Life Technologies, Carlsbad, CA, USA, cat. no. 11140050)

L-Glutamine (GIBCO, Life Technologies, Carlsbad, CA, USA, cat. no. 25030024)

Fetal Calf Serum (GIBCO, Life Technologies, Carlsbad, CA, USA, cat. no. 10500064)

DMEM + pyruvate medium (GIBCO, Life Technologies, Carlsbad, CA, USA, cat. no. 41966029)

Lenti-X™ concentrator (Takara, Shiga, Japan, cat. no. 631231)

Polyethylenimine (PEI)

Polybrene (Sigma aldrich, St. Louis, MO, USA, cat. no. TR-1003-G)

### 2.2. Equipment

Conical 15 mL tubes (Greiner Bio-One, Frickenhausen, Germany, cat. no. 188271)

Conical 50 mL tubes (Greiner Bio-One, Frickenhausen, Germany, cat. no. 227661)

Cell culture dishes, 145/20 mm (Greiner Bio-One, Frickenhausen, Germany, cat. no. 639160)

24 well plates (Greiner Bio-One, Frickenhausen, Germany, cat. no. 662 160)

Glass Pasteur pipettes (VWR International Ltd., Leicester, UK, cat. no. 612-1701)

70 μm cell strainer (Corning, Falcon, NY, USA, cat. no. 352350)

Disposable scalpels (Swann-Morton, Sheffield, UK, code 0501)

Centrifuge (Eppendorf, Hamburg, Germany, cat. no. 5810R)

CO_2_ incubator (5% CO_2_, 37 °C)

FLUOstar Omega microplate reader

Level 2 biosafety cabinet

37 °C shaking platform

Fluorescence microscope (DM IL LED inverted microscope, Leica, Germany)

Flow cytometer (SH800 cell sorter, Sony, Japan)

### 2.3. Transfer Plasmid

The lentiviral transfer plasmid eGFP-ffLuc_epHIV7 contains the eukaryotic translation elongation factor 1 α (EF-1α) promoter, driving the expression of enhanced green fluorescent protein (eGFP) and firefly luciferase (ffLuc) reporter genes (Supplementary Figure S1a). The EF-1α promoter offers a more stable and high-level eGFP-ffLuc transgene expression in long-term cultures with minimal silencing activity (Supplementary Figure S1b) [14]. eGFP provides a selection tool to sort positive clones as well as visual monitoring of ffLuc transgene expression. The luciferase reporter provides a bright and stable bioluminescence signal by oxygenation of its substrate D-luciferin as a sensitive method for cell viability assays. All plasmids used in the study are deposited and made available for public use via the non-profit Addgene plasmid repository under the Uniform Biological Material Transfer Agreement (UBMTA; Table 1).

### 2.4. Cell Culture Reagents

#### 2.4.1. Human Neuroblastoma Tissue Acquisition

Samples were included in the study after obtaining informed consent of the parents of the neuroblastoma patients and approval from the ethical committee at the Princess Maxima Centrum (PMC). Freshly resected neuroblastoma biopsies were collected and processed immediately following histological confirmation of the presence of viable tumour tissue. Samples were either diagnostic from the primary site, metastatic, or relapsed.

#### 2.4.2. Neuroblastoma Tissue Processing

Tumour tissues were minced into 3–4 mm pieces, snap-frozen in liquid N2 and then stored at −80 °C while other pieces were saved for fixation in 4% paraformaldehyde and stored at −20 °C for 3D imaging. The remainder was digested using collagenase type I, II, and IV (2 mg/mL) in the optimized culture medium by 2 h agitation at 37 °C. Undigested tissue fragments were removed by passing through a sterile 70 μm cell strainer. Resulting suspensions containing small tumour fragments were washed with cold organoid medium followed by centrifugation at 1050 RPM for 5 min at 4 °C. Optionally, in the presence of highly visible red pellets, 2 mL of red blood cell lysis buffer may be used to lyse erythrocytes by 10 min agitation at room temperature. Cells were washed again, followed by centrifugation at 1050 RPM for 5 min at 4 °C. Half of the tumour fragments were cultured in neuroblastoma organoid medium while the other half were cultured in neuroblastoma organoid medium with 20% human plasma.

#### 2.4.3. Neuroblastoma Organoid Medium

Neuroblastoma organoid medium consists of Dulbecco’s modified Eagle’s medium (DMEM) with low glucose and Glutamax™ supplement, supplemented with 25% Ham’s F-12 Nutrient Mix, B-27 supplement minus vitamin A (50×), N-2 supplement (100×), 100 U/mL penicillin, 100 μg/mL streptomycin, 20 ng/mL animal-free recombinant human EGF, 40 ng/mL recombinant human FGF-basic, 200 ng/mL recombinant human IGF-I, 10 ng/mL recombinant human PDGF-AA and 10 ng/mL recombinant human PDGF-BB.

#### 2.4.4. DIPG Organoid Medium

HSJD-DIPG-007 cell tumour stem medium (TSM) consists of 48% Neurobasal-A medium, 48% DMEM/F-12, 1% Glutamax, 1% MEM non-essential amino acid solution, 1% HEPES buffer solution, 1% sodium pyruvate and 0.2% primocin. The TSM was supplemented with 2% B27 supplement minus vitamin A, 20 ng/mL recombinant human FGF-2, 20 ng/mL recombinant human EGF, 10 ng/mL recombinant human PDGF-AA, 10 ng/mL recombinant human PDGF-BB, and 2 µg/mL heparin.

#### 2.4.5. Neuroblastoma Cell Culture

Patient-derived neuroblastoma organoids were previously established [4], (Kholosy et al., unpublished). Cells were cultured in organoid medium in a 37 °C incubator with a humidified atmosphere of 5% CO_2_. The medium was refreshed twice a week. The medium was refreshed by removing the spent cell culture media into a falcon tube and adding half of the recommended volume of fresh culture medium to the culture flask. The cell suspension was centrifuged at 300× *g* for 5 min at room temperature, and the supernatant was discarded. When the cells were growing in big spheres, the spheres were disrupted by resuspending the cells in Accutase and pipetting the cells up and down with a P1000 pipette with a P1000 filter tip and a P200 pipette tip on top. The cells were washed with plain medium (DMEM-GlutaMAX) and resuspended in half of the organoid medium and transferred to the culture flask. All organoids were proven mycoplasma free after routine testing.

#### 2.4.6. DIPG Cell Culture

HSJD-DIPG-007 cells were collected from a glioblastoma multiforme (WHO grade IV) during autopsy and shown to retain the tumour-specific mutations H3.3-K27M and ACVR1-R206H [15]. Cells have successfully been used for the development of a well-established in vitro 3D neurospheres model [16,17,18] and in vivo orthotopic xenograft model [19,20,21,22]. Neurospheres were grown in serum-free TSM in a humidified incubator with 5% CO_2_ at 37 °C. HSJD-DIPG-007 cells were mycoplasma free after routine screens.

#### 2.4.7. HEK293 Cell Culture

HEK293T cells were cultured in medium containing DMEM + pyruvate medium (DMEM + 4.5 g/L D-Glucose, L-Glutamine, 10% Fetal Calf Serum, two mM L-Glutamine, 100 U/mL, Penicillin with 100 mg/mL Streptomycin and Non-Essential Amino Acids).

### 2.5. Statistics

Differences in the ex vivo assays were determined by two-tailed Student’s t-test. Findings were considered statistically significant when p-values were lower than 0.01. Results were expressed as the mean and standard error of the mean (SEM). Linear regressions and regression values (R) were computed and plotted using the “regression” function included in MATLAB^®^ R2016a data processing software (Matlab, Mathworks, Natick, MA, USA).

### 2.6. Methods

#### 2.6.1. Part 1: Virus Production

##### Virus Production—Day 0

1. Seed HEK293T cells in a 145/20 mm cell culture dish in 20 mL HEK293 medium to achieve 60–80% confluency the day after and place them in a CO_2_ incubator (5% CO_2_, 37 °C).

##### Virus Production—Day 1

1. Four hours prior to transfection, refresh the HEK293T medium without detaching the adherent cells by removing 20 mL medium via the sides of the dish and adding 15 mL fresh medium.
2. Transfer 315 μL of PEI (1 mg/mL) to a 15-mL conical tube containing 2 mL Opti-MEM and mix well by brief vortexing prior to 5 min incubation at RT.
3. Transfer plasmid DNA (7.2 μg pHDMG (ENV), 3.6 μg pRC/CMV-rev1b, 3.6 μg pHDM Tat1b, 3.6 μg pHDM Hgpm2 and 45 μg of the transfer plasmid) to a 15 mL conical tube containing 3 mL Opti-MEM and mix well by brief vortexing.
4. Add PEI dropwise to plasmid DNA and mix well by brief vortexing prior to 30 min incubation at RT.
5. Transfer the PEI/DNA mixture dropwise to the cells and gently rotate the plate to equally distribute the PEI/plasmid DNA mixture in the plate and incubate the plate overnight in a CO_2_ incubator (5% CO_2_, 37 °C).

##### Virus Production—Day 2

6. Replace HEK293T medium with 20 mL fresh neuroblastoma organoid medium without detaching the adherent cells.

##### Virus Production—Day 3

7. Transfer all medium from the plate into a 50 mL conical tube for storage at 4 °C until further use in step 10.
8. Add 20 mL fresh neuroblastoma organoid medium to the HEK293T cells and incubate the cells for another 24 h.

##### Virus Production—Day 4

9. Remove all medium from the plate and transfer it into the same collecting tube used to harvest the previous day.
10. Pass the harvested medium through a 0.45 mm filter to remove any cell debris. Note: use only low protein binding filters such as cellulose acetate or polyethersulfone (PES).

#### 2.6.2. Part 2: Concentrating Lentiviral Supernatants

##### Concentrating Lentiviral Supernatants—Day 0

11. Add 10 mL of Lenti-X Concentrator to the clarified virus supernatant.
12. Mix gently by inversion and incubate at 4 °C for 24 h.

##### Concentrating Lentiviral Supernatants—Day 1

13. Centrifuge the tube at 1500× *g* for 45 min at 4 °C.
14. Carefully aspirate and discard the supernatant from the tube without disturbing the off-white pellet.
15. Resuspend the pellet in 500 μL neuroblastoma organoid medium.
16. Store the concentrated virus at −80 °C in 50 μL aliquots for single use.

#### 2.6.3. Part 3: Virus Transduction of Patient-Derived Cells

##### Lentiviral Transduction of Neuroblastoma and DIPG Organoids—Day 0

17. Take a T25 flask containing the organoids out of the incubator, disperse the culture medium by pipetting the medium several times over the T25 flask culture layer surface and transfer the medium into a 15 mL conical tube.
18. Centrifuge the 15 mL conical tube at 300× *g* for 5 min.
19. Aspirate the supernatant, resuspend and dissociate the cell pellet in 150 μL medium (neuroblastoma organoid medium or TSM for DIPG organoids) containing 10 μg/mL polybrene.
20. Add 50 μL virus to the cell suspense and mix well.
21. Transfer the cell–virus mixture into a 48-well plate and cover the plate with parafilm.
22. Spinoculation step: Centrifuge the 48-well plate at 600× *g* for 1 h at 32 °C.

Note: The break speed of the centrifuge is 0.

23. Transfer the 48-well plate into the incubator for 2–3 h.

Note: To prevent evaporation, add medium/PBS into the wells surrounding the well containing the cell–virus mixture before transferring the 48-well plate into the incubator.

24. Add 300 μL of culture medium (neuroblastoma organoid medium or TSM for DIPG organoids) to the well and transfer the 48-well plate into the incubator.

##### Lentiviral Transduction of Neuroblastoma and DIPG Organoids—Day 1

25. Disperse the culture medium by pipetting several times over the 48-well surface and transfer the medium into a 15 mL conical tube.
26. Centrifuge the 15 mL conical tube at 300× *g* for 5 min.
27. Aspirate the supernatant and resuspend the cell pellet in medium (neuroblastoma organoid medium or TSM for DIPG organoids).
28. Transfer the cell suspension into a new T25 culture flask and incubate the cells for further expansion.

#### 2.6.4. Part 4: Purification Transduced Cells by FACS

Transduced primary neuroblastoma and DIPG cells will start expressing eGFP proteins 1–2 days after transduction. Cells were expanded for x days, and the brightest eGFP expressing cells were selected out and sorted if necessary by FACS using the following steps:

29. Transfer the transduced and matching wild type primary cells in separate 15 mL conical tubes.
30. Dissociate the cells with 1 mL Accutase until single-cell suspensions are obtained (check under the microscope).
31. Add 10 mL of serum-free culture medium to halt the dissociation reaction.
32. Centrifuge the tubes at 300 RCF for 5 min at 4 °C.
33. Aspirate and discard the supernatants.
34. Add 1 mL of serum-free culture medium to the tubes.
35. Transfer the cells into Falcon^®^ Round-Bottom Tubes with Cell Strainer Cap.
36. Keep all tubes on ice and covered with aluminium foil to protect from light.
37. All samples are analysed and sorted on a Sony SH800 Cell Sorter.

#### 2.6.5. Part 5: Antibody-Dependent Cellular Cytotoxicity (ADCC) Assay

##### Organoid Seeding—Day 0

38. Harvest the organoids from cell culture and transfer the organoid suspensions into a 50 mL conical tube.
39. Spin the tube at 300× *g* for 5 min.
40. Discard the supernatant medium and resuspend the organoid pellet in 1 mL Accutase.
41. Dissociate the organoids into single cells by pipetting up and down against the bottom of the 50 mL tubes.

Note: Place a P100 (without filter) tip on top of a P1000 pipette tip to mechanically separate the organoids. Pipet at least 20 times up and down. Check under a microscope whether all spheres have been dissociated into predominantly single cells. When large spheres are still present, repeat the pipetting procedure.

When the cell suspension contains predominantly single cells, continue with the next steps:

42. Add 9 mL organoid medium to inactivate the Accutase and mix the solution by pipetting up and down.
43. Spin the tube at 300× *g* for 5 min.
44. Remove the supernatant medium using a suction pipette.
45. Resuspend the cell pellet in 1 mL organoid medium
46. Filter the single-cell organoid suspension by transferring the sample to FACS tubes.
47. Count the cells by mixing 10 µL of the single-cell suspension with 10 µL Trypan blue. Transfer 10 µL of the mixture into the counting slides on both sides A and B and count the cells.
48. Calculate the required volume to seed the optimal number of single cells (in this study, 40,000 cells/well) in 150 µL medium/well into a white 96-well plate.

Note: leave some wells to fill them with medium only (without cells) to correct for background signals and add PBS to the unused wells surrounding the sample-containing wells.

49. Add 200 µL of organoid medium into the designated wells containing medium only.

##### Dinutuximab Treatment Neuroblastoma Organoids—Day 0

50. For cytotoxicity studies, NB organoid lines were not treated in the absence or presence of PBMCs (controls) or treated with dinutuximab in the presence of PBMCs.
51. Add different concentrations of dinutuximab (10 µg/mL, 1 µg/mL, 100 ng/mL) to the neuroblastoma cells.
52. Place the 96-well plate into the incubator until the isolated PBMC cells are added.

##### Peripheral Blood Mononuclear Cell (PBMC) Isolation from Donor Blood—Day 0

53. PBMCs were isolated from the blood of healthy donors using the Lymphoprep Kit (Stemcell Technologies) according to the manufacturer’s protocol.
54. Isolated PBMCs washed with an equal amount of PBS + 2% FBS and resuspend the PBMC pellet in 2 mL organoid medium.
55. Count the PBMCs and calculate the number of PBMCs needed to obtain the desired effector to target (E:T) ratio (here we used 20:1 E:T ratio, 800.000 PBMCs in 50 μL organoid medium per well).

##### Coculture of Organoids and PBMC—Day 0

56. Add 800,000 PBMCs in 50 µL organoid medium to each requested well.
57. Incubate the plate for 4 h in the incubator.
58. Add D-luciferin (1:200 dilution) to the wells.
59. Spin the plate at 300× *g* for 5 min.
60. Incubate the plate for 5 min in the incubator.
61. Wrap the plate in aluminium foil to protect from light and read out the bioluminescence signal on the plate reader (FLUOstar Omega).

Note: Firefly luciferin is sensitive to light and must be protected and stored in the dark. GD2 expression levels were previously assessed for all organoid lines by means of flow cytometry (Supplementary Figure S2).

## 3. Results

### 3.1. Transduction Efficiency of DIPG Neurospheres and Neuroblastoma Organoids

**Figure 1:**
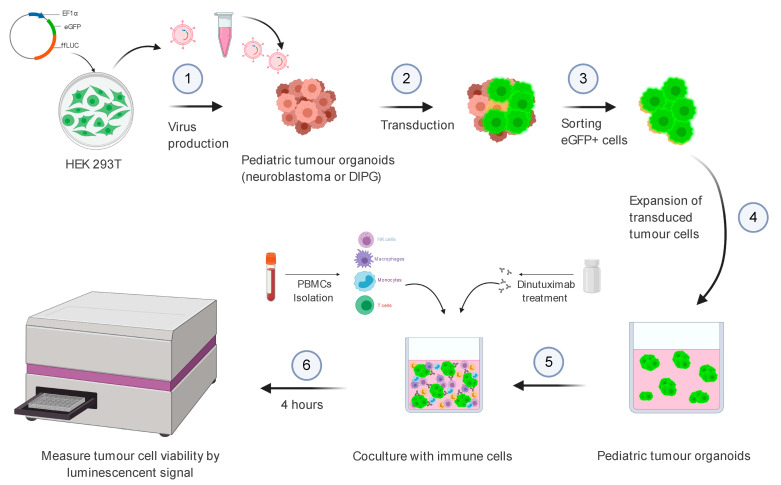
Schematic representation of study design and experimental workflow. Paediatric tumour organoids (neuroblastoma and DIPG organoids) were transduced with a lentiviral vector carrying eGFP-ffLuc fusion reporter genes. eGFP expressing cells were sorted and expanded for coculture assays. Paediatric organoids were cocultured with allogenic PBMCs with/without dinutuximab treatment. The tumour cell viability was measured using the ADCC luciferase assay after the administration of D-Luciferin.

**Figure 2:**
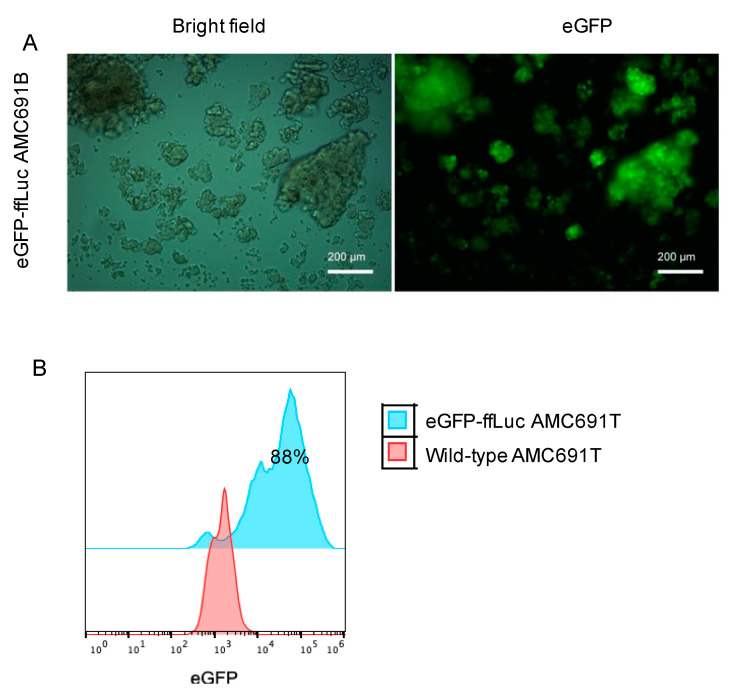
Patient-derived neuroblastoma organoids were examined for GFP expression 96 h after lentiviral transduction. (**A**) Fluorescence microscopy of patient derived neuroblastoma organoid AMC691B 96 h after infection after lentiviral transduction showing bright field (**left**) and fluorescence (**right**) images. (**B**) Flow cytometry analysis of one patient derived neuroblastoma organoid (AMC691T) after lentiviral transduction. Proportions of AMC691T.

**Figure 3:**
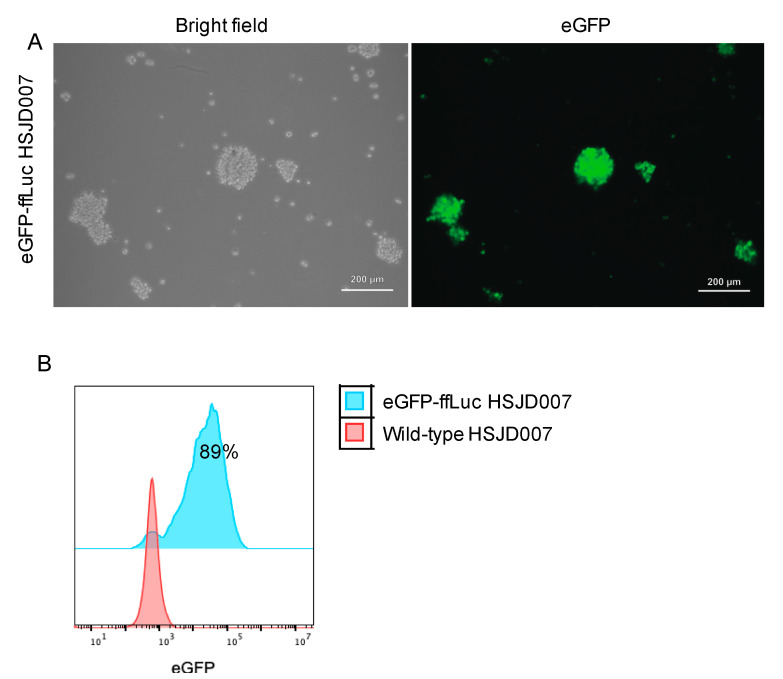
Patient-derived DIPG neurospheres were examined for GFP expression 96 h after lentiviral transduction. (**A**) Fluorescence microscopy of patient-derived DIPG neurospheres 96 h after infection after lentiviral transduction showing bright field (**left**) and fluorescence (**right**) images. (**B**) Flow cytometry analysis of patient-derived DIPG neurospheres (HSJD007) after lentiviral transduction. Proportions of HSJD007 expressing eGFP (blue) were detected using flow cytometry 96 h following lentiviral vector transduction compared to wild type counterparts (red).

To assess the transduction efficiency of our protocol, eGFP expression in the transduced tumour organoids was analysed using fluorescence microscopy and flow cytometry as described in Figure 1. The onset of eGFP expression in both neuroblastoma organoids and DIPG neurospheres occurred 48 h after transduction. Monitoring of eGFP expression reflected by the fluorescence signal intensity showed a maximum expression at 3 to 4 days (data not shown) after virus transduction. Based on flow cytometry, the transduction efficiency was higher than 30% in all tumour organoids (Figure 2 and Figure 3). eGFP positive cells were selected using FACS when transduction efficiency is lower than 70%. Patient-derived neuroblastoma organoids and classical neuroblastoma cell lines exhibited a transduction efficiency from 30% to 94% (Figure 2; Supplementary Figures S3 and S4). DIPG cells displayed a transduction efficiency greater than 88% determined by flow cytometric measurement of eGFP fluorescence intensity (Figure 3). For all transduced cells, the eGFP intensity corroborated the estimates found in the fluorescence micrographs (Figure 2 and Figure 3). Together, our data demonstrate the efficiency and robustness of our method for generating stably transduced patient-derived organoids from different paediatric cancer types using serum-free culture protocol.

### 3.2. Evaluation of the Correlation between Bioluminescence Signal and the Number of Living Cells for DIPG Neurospheres and Neuroblastoma Organoids

**Figure 4:**
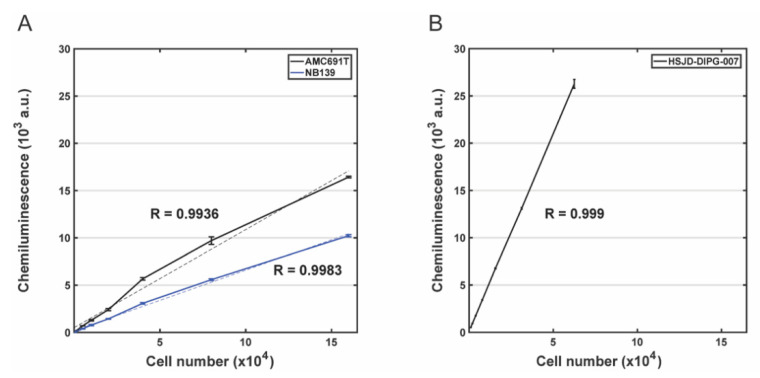
Correlation of tumour cell number and chemoluminescence signal using the luciferase assay for in vitro cell proliferation measurement. (**A**) Measurement of luminescence in two transduced eGFPffLuc neuroblastoma organoids (NB139 and AMC691T). (**B**) Measurement of luminescence in two transduced eGFP-ffLuc DIPG neurospheres (HSJD007). Different cell densities were seeded onto 96-well plates and the luminescence was measured after 10 min in their presence. Results are represented by average of triplicate values. R shows the linear correlation of luminescence and cell number in neuroblastoma and DIPG cells.

Transduced neuroblastoma and DIPG cells exhibited a bioluminescence signal that was found to be linearly proportional to their seeding densities, as indicated by the correlation coefficient (R) greater than 0.99 in all transduced cell lines tested, 0.999 in HSJD-DIPG-007 cells (Figure 4a), 0.9936 in AMC691T+ cells and 0.9983 in NB139+ cells (Figure 4b). Positive correlations indicate that eGFP-ffLuc expression directly reflects the number of neuroblastoma or DIPG cells in vitro. Moreover, bioluminescence signals could be detected at the lowest tested seeding densities, i.e., 5000 cells for neuroblastoma cells and 1000 cells for HSJD-DIPG-007 cells, with mean signals 299-fold (NB139+), 390-fold (AMC691T+) and 47-fold (HSJD007+) greater than those found for density-matched non-transduced cells (Figure 4, Supplementary Figure S5). Our data demonstrate the sensitivity of our luciferase-based system for measuring the viability of transduced patient-derived DIPG and neuroblastoma organoids in vitro.

### 3.3. Bioluminescence-Based Ex Vivo Test Coculture System of Pediatric Tumour Organoids and PBMCs for Assessment of Anti-GD2 Immunotherapy

**Figure 5:**
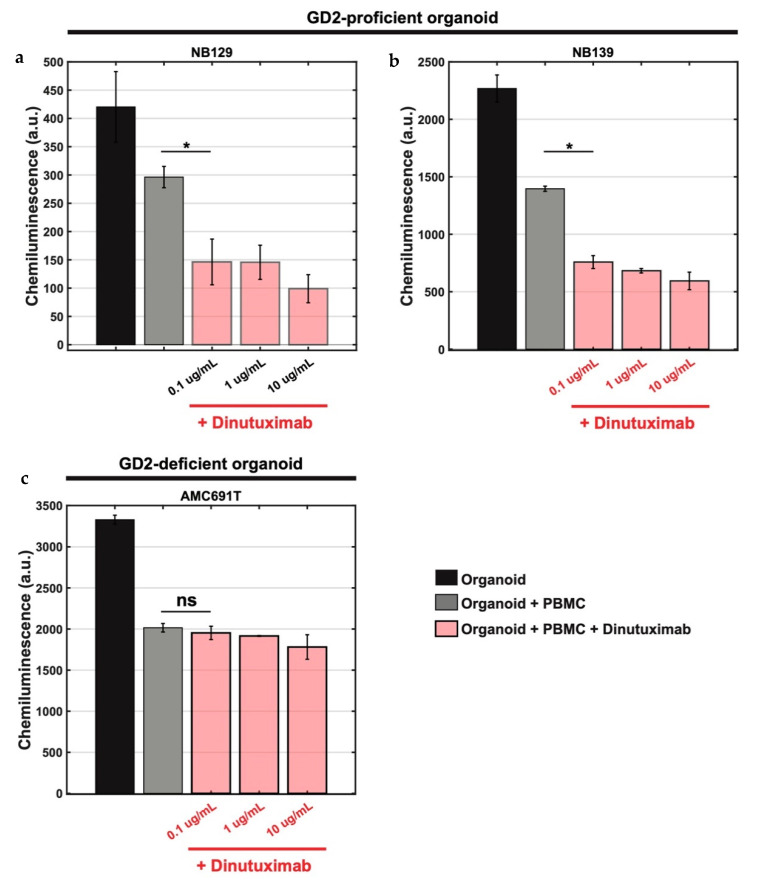
Effect of Dinutuximab treatment on neuroblastoma cells cocultured with PBMCs. Chemiluminescence-based cell viability was measured using the ADCC luciferase assay. (**a**,**b**) The viability of neuroblastoma cells (black column) were compared with cocultured neuroblastoma cells with PBMCs (grey column, used as negative control) in the presence of dinutuximab, an FDA-approved monoclonal antibody targeting GD2 antigen. Tumour cells were exposed for 4 h to 0.1, 1 and 10 ug/mL of dinutuximab in GD2-proficient organoid lines NB129 and NB139. (**c**) Similar to a and b, the neuroblastoma organoids (cocultured with PBMCs) were exposed for 4 h to 0.1, 1 and 10 ug/mL of dinutuximab in GD2-deficient organoid lines AMC691T cells. All experiments were performed in triplicate. Error bars represent standard deviation values. * *p* value is less than 0.01.

We investigated whether our system can be applied in a coculture system to study ex vivo drug effects on immune-mediated tumour cell killing. As a proof-of-concept, eGFP-ffLuc-transduced neuroblastoma organoid cells were cocultured with freshly isolated healthy donor PBMCs to study the ex vivo effects of dinutuximab, an FDA-approved monoclonal antibody targeting GD2 expressed on the surface of most neuroblastoma cells. Immune cell-mediated cytotoxic effects of dinutuximab were studied on three transduced neuroblastoma organoids: NB129, NB139 (GD2 expressing organoids, Supplementary Figure S2) and AMC691T (GD2 deficient organoids, Supplementary Figure S2). These neuroblastoma organoid lines are HLA-ABC positive, as shown by flow cytometry analysis (Supplementary Figure S2). PBMCs alone (control) exhibited a considerable cytotoxic effect when cocultured with neuroblastoma cells (Figure 5). However, the combination of dinutuximab with freshly isolated PBMCs, using a 20:1 effector to tumour (E:T) ratio, induced a significant additional decrease in cell viability of GD2 proficient NB organoids NB139 cells and NB129, which achieving specific lysis of more than 50% at the same E:T ratio of 20:1 (*p* < 0.01) (Figure 5a,b). Conversely, no significant additional effect of dinutuximab on the cytotoxicity of PBMCs on GD2 deficient AMC691T organoids was observed, confirming the GD2-specific cytotoxicity of dinutuximab against neuroblastoma organoids (Figure 5c). Our findings demonstrate the potency of our organoid-PBMC based coculture system for personalized drug testing and the cytotoxic effect of dinutuximab on GD2 proficient NB cells (NB 139 and NB129) when combined with freshly isolated allogeneic PBMCs.

## 4. Discussion

Most of our knowledge in immuno-oncology is currently gathered from traditional 2D tumour cell cultures and mouse models [23,24]. However, traditional tumour cell lines fail to recapitulate the heterogeneity and morphology of human cancers, and the mouse immune system is very different from the human immune system [23,24]. Together with the rapid increase in the development of novel promising therapeutics in the field of paediatric immuno-oncology, this makes the development of novel, more representative model systems for immunotherapy testing crucial.

In the present study, we describe the development of coculture models of paediatric tumour organoids and immune cells for ex vivo bioluminescence-based cytotoxicity studies. Patient-derived organoids represent highly valuable models in translational research for drug testing and have successfully been used in tumour-immune cell cultures for studying cancer-immune cell interactions [23,24]. To circumvent the difficulty of generating stable eGFP and ffLuc transgenes expression in primary cell lines from paediatric tumour tissue, we devised an efficient, serum-free lentiviral transduction protocol in patient-derived paediatric tumour organoids. This transduction protocol was applied in two patient-derived paediatric tumours from different tissue types, i.e., neuroblastoma organoids and DIPG neurospheres. We incorporated eGFP-ffLuc transgenes, allowing the transduced cells to exhibit fluorescence and bioluminescence properties. We evidenced that this photon emission can be used as a fingerprint of tumour growth in all neuroblastoma organoids and HSJD-DIPG-007 neurospheres studied here. Optical properties of the latter tumour model are of particular importance, as its intracerebral engraftment to generate the orthotopic model in vivo makes the monitoring of tumour growth with instruments, like a calliper, impossible.

## 5. Comparison with Other Methods

A key advantage of our method is that the lentiviral transduction protocol used leads to efficient, stable transgene expression, and that successful transduced primary paediatric tumour lines can be generated and stored. Our protocol further used serum-free culture conditions, while other protocols use either serum-rich or serum-reduced medium [25]. Patient-derived organoids are preferably cultured in animal serum-free medium with defined recombinant growth factors because the presence of serum and undefined growth conditions may contain differentiation factors causing growth arrest related to differentiation for some neuronal cultures. In addition, instead of using ultracentrifugation to concentrate the lentiviral stock, we used Lenti-X Concentrator as a faster and more efficient method to enhance the lentiviral titer by up to 100-fold in less than 1 h. Another difference is that our protocol utilizes cell sorting to enrich the high-expressing population of transduced tumour cells. Since the expression of firefly luciferase is linked to eGFP expression, the expression of luminescence signals and the expression of firefly luciferase can be controlled by selecting and sorted cells with the desired intensity of eGFP expression. In addition, since the promoter EF1 is a human-derived promoter, it has a minimal chance of gene silencing compared to other promoters used in other protocols. In terms of transduction efficiency, here, to the best of our knowledge, efficient lentiviral transduction of NB organoids has not been reported before. For DIPG, the yields obtained here were found to be significantly greater than those reported in past studies using similar primary/patient-derived paediatric tumour organoids [25].

Conventional metabolic-based viability assays such as the CellTiterGlo^®^ luminescent cell viability assay cannot discriminate between the metabolic activities of different cell types [26,27], making these assays incompatible for cell viability studies in tumour-immune cell cocultures. In contrast, luciferase-induced luminescence and GFP-induced fluorescence signals generated by our method accurately reflect changes in tumour cell number as luciferase and GFP are endogenously expressed in tumour cells exclusively. Radioactive chromium ((51) Cr) release and calcein acetoxymethyl ester (CAM) assays have been extensively used for in vitro assessment of antibody-dependent cell-mediated cytotoxicity (ADCC). Several disadvantages of this assay include handling of radioactive compounds, limited sensitivity due to spontaneous leakage, and their incompatibility for large scale high-throughput screening (HTS), which gives our approach an advantage due to the absence of these limitations [8,9].

## 6. Biosafety Considerations

Our method involves the transduction of patient-derived tumour tissue using lentiviral vectors. Handling lentiviral expression systems requires biosafety level 2 practices and following the institutional and national guidelines concerning biosafety is necessary. Although we used a third-generation lentiviral vector in this protocol, which is known to have an improved safety profile compared to the second-generation vector [28], risk reduction measures have to be routinely undertaken during all lentiviral handling steps.

## 7. Technical Limitations

It is important to note that the use of lentiviral vectors to generate long-term eGFP-ffLuc transgene expression implicates two main consequences. First, since high transgene levels may have a negative influence on the growth rates of primary tumour cells, rapidly growing tumour cells due to low transgene expression may eventually be selected out and take over the culture. This might result in the production of lower bioluminescence and fluorescence signals. Second, although the transduction was successful in all attempted tumour organoids and cell lines, the intensity of transgene expression was variable between different tumour lines due to differences in transduction efficiency. Therefore, further individual optimization steps are needed to identify the minimum number of cells that gives a reasonable bioluminescence intensity. Experimental conditions such as the E:T ratios, and E:T incubation times will vary based on cell types used and should be individually optimized for a particular assay.

## 8. Applications of the Protocol

Neuroblastoma cells express high levels of gangliosides and sialic acid-derived carbohydrates and proteins. These carbohydrate molecules play a crucial role in the aggressive phenotype of the disease, including invasion, migration, metastasis and adhesion [29]. One of these epitopes, ganglioside GD2, is an oncofetal antigen expressed mainly in the fetal adrenal cells as well as mature cells such as peripheral neurons and skin cells [30,31]. Despite being poorly immunogenic epitopes, targeting ganglioside GD2 using anti-GD2 IgG MAbs in combination with interleukin-2 (IL-2), granulocyte-macrophage colony-stimulating factor (GM-CSF) and 13-cis-retinoic acid resulted in a significant improvement in neuroblastoma patient survival [2,32]. However, the clinical efficacy has been seen in patients with minimal residual disease, not with bulky neuroblastomas [32]. Besides, the survival rates of high-risk neuroblastoma patients are still far behind other malignant diseases in paediatric oncology; therefore, new combination approaches with dinutuximab are urgently needed.

One potential application of this system is their anticipated use to measure ADCC in a high-throughput fashion. Our study reports an easy to follow workflow to generate an ex vivo coculture system that can be easily integrated into high-throughput drug screenings to identify potential drug combinations with dinutuximab. Combination studies may include targeted drugs as well as other immunotherapeutic drugs such as immune checkpoint inhibitors. Approaches such as CAR-T cells, TCR T cells and gamma delta T cells represent an emerging therapeutic strategy to treat a variety of cancers including neuroblastoma and DIPG. The same platform can be used in precision medicine programs in paediatric oncology by coculturing patient-derived paediatric tumours with autologous PBMCs, thereby perhaps allowing for the prediction of the effectivity of immunotherapeutics in a patient-dependent fashion (Supplementary Video S1). Other applications of the protocol may include the generation of patient-derived xenograft (PDX) or humanized mouse models using the labelled organoids for in vivo testing of therapeutic agents as it allows us to visualize and monitor tumour progression in real-time. Lastly, similar approaches can be used for cocultures of patient-derived cancer cells with other cell types such as fibroblasts and mesenchymal stem cells or particular wild type/engineered immune cell types.

## Figures and Tables

**Table 1:** Plasmids used in the study.

Name of Transgene	Vector	Description
FfLuc, eGFP	pJ01668	epHIV7 vector, firefly luciferase, T2A sequence, eGFP
	pHDM-Hgpm2	Packaging plasmid for lentiviral vector
	pRC/CMV-rev1b	Regulatory plasmid for lentiviral vector
	pHDMG	Envelope plasmid used for lentivirus production
	pHDM-tat1b	Regulatory plasmid for lentiviral vector

## Supplementary Materials

The following are available online at https://www.mdpi.com/article/10.3390/jpm11090869/s1, Figure S1: Schematic representation of the transfer plasmid used in the study. Figure S2: Flow cytometric staining for three analysed tumour samples (NB129, NB139 and AMC691T) showing the expression of GD2 and HLA-ABC, Proportions of the classical neuroblastoma cell line. Figure S3: KCNR cells expressing GFP were detected using flow cytometry, 5–7 days following lentiviral vector transduction compared to wild type counterparts. Figure S4: Proportions of neuroblastoma organoids AMC691T, NB129 and NB139 expressing GFP were detected using flow cytometry. Figure S5: Effect of Dinutuximab treatment on neuroblastoma cells cocultured with PBMCs. Video S1: Coculture of GFP-ffLuc autologous NB tumour NB139 with peripheral blood T cells.
