# Supplementary material for: Neuroblastoma and DIPG Organoid Coculture System for Personalized Assessment of Novel Anticancer Immunotherapies
Source: J Pers Med. 2021 Aug 30;11(9):869. doi: 10.3390/jpm11090869 (PMC8466534; doi:10.3390/jpm11090869)
Supplement: Supplementary file 1 [file jpm-11-00869-s001.zip › jpm-1366743-supplementary/jpm-1366743-supplementary.pdf]

Journal of Personalized Medicine

MDPI

Supplementary Figures

A

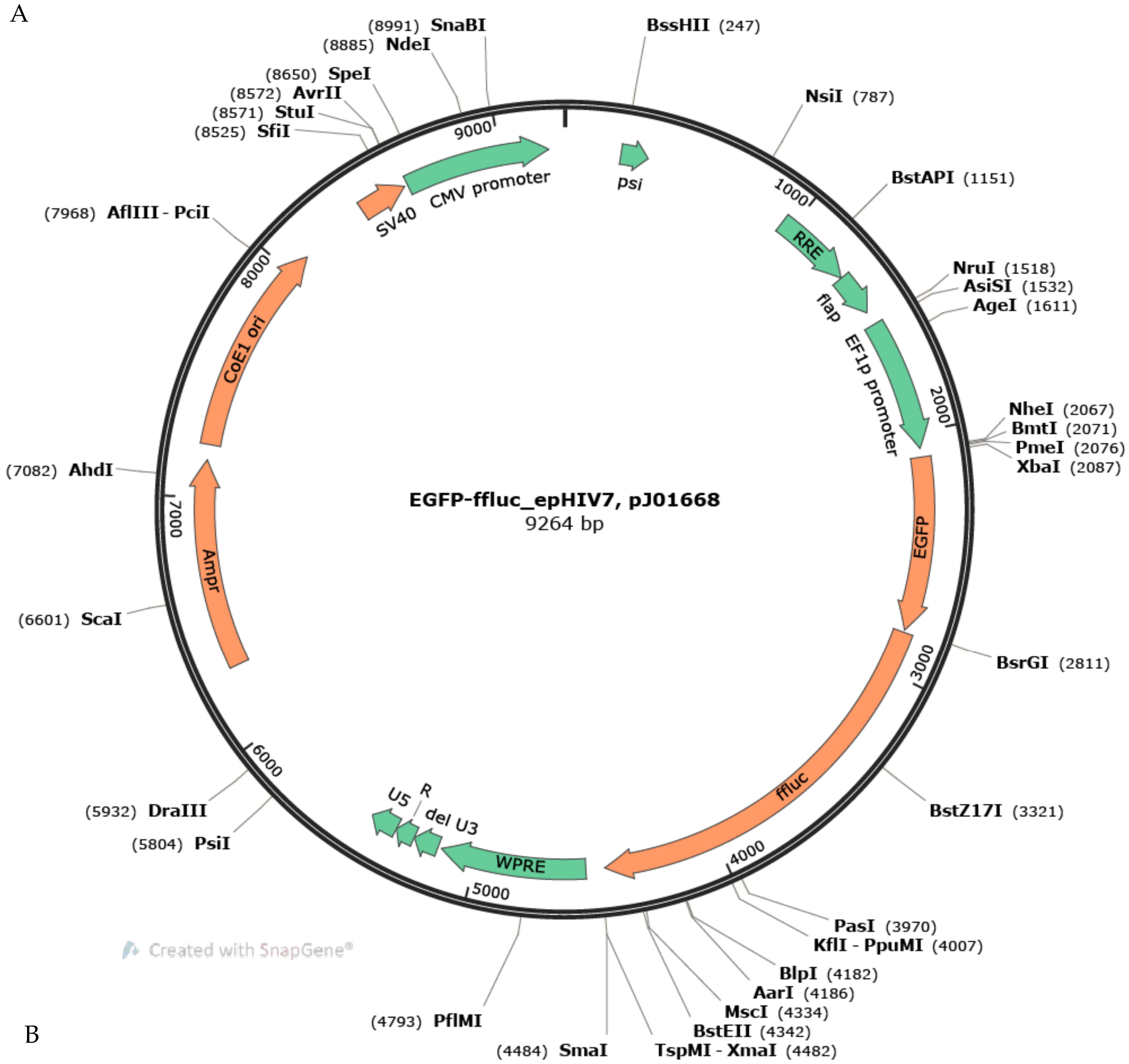

B

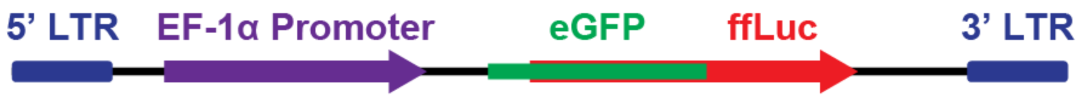

Supplementary Figure S1. Schematic representation of the transfer plasmid used in the study. A) Plasmid map (9,264 base pairs). B) Both reporter genes eGFP and firefly luciferase (ffLuc) are under the control of the elongation factor 1α (EF-1α) promoter.

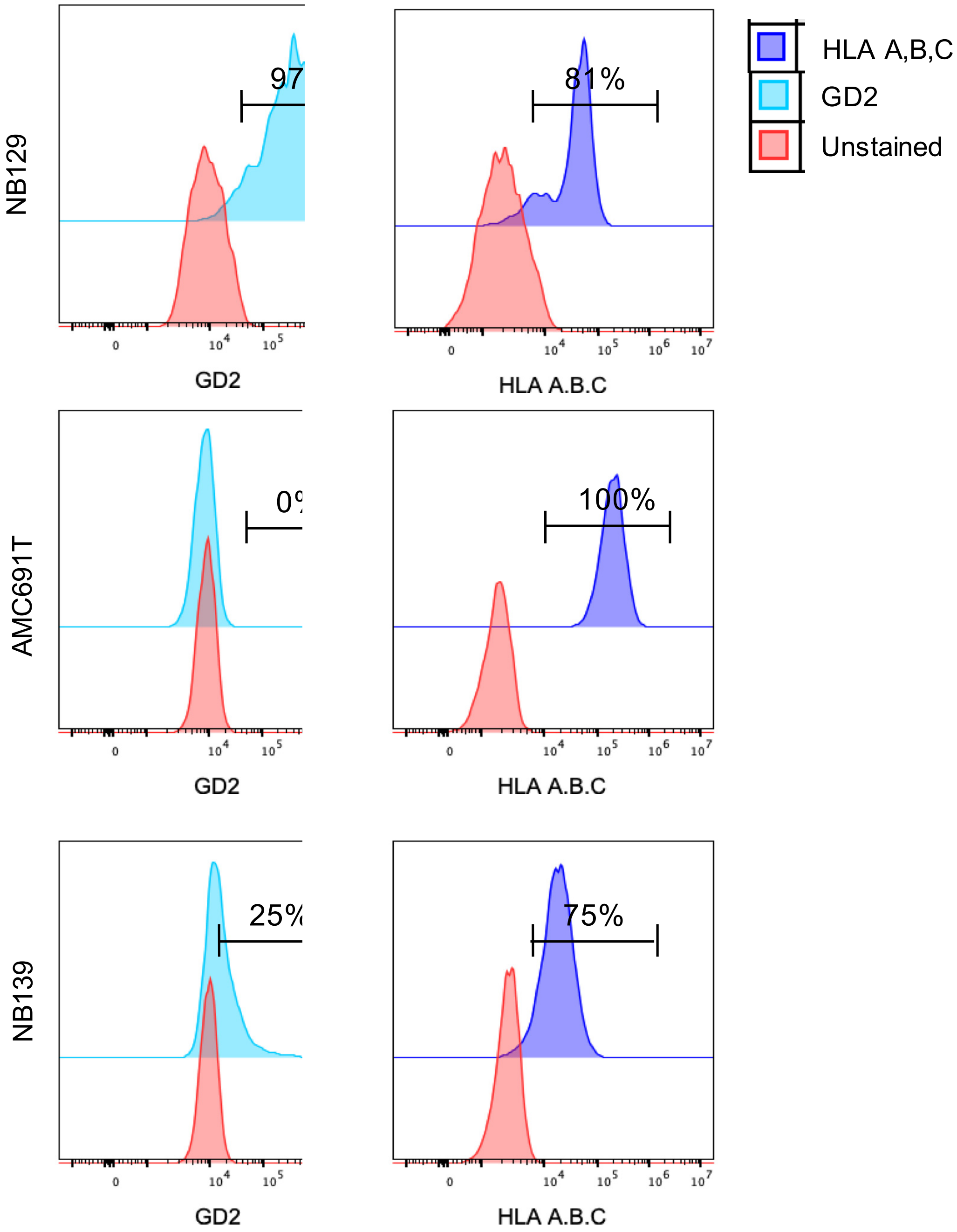

Supplementary Figure S2. Flow cytometric staining for three analysed tumour samples (NB129, NB139 and AMC691T) showing the expression of GD2 and HLA-ABC.

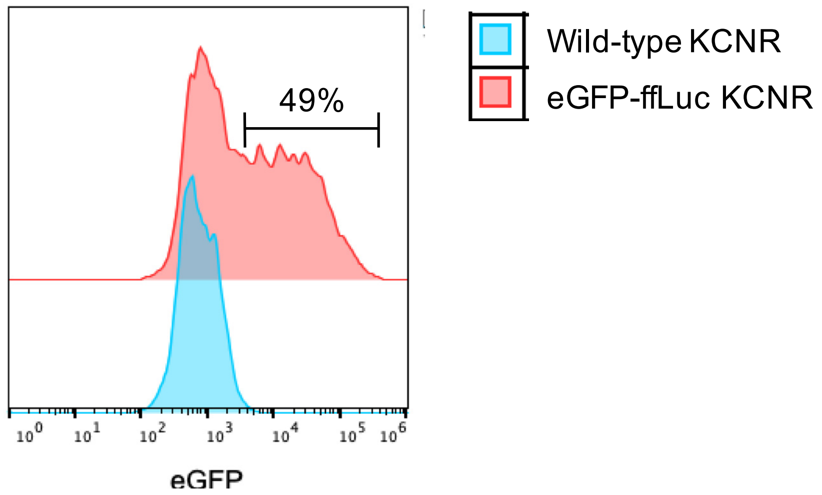

Supplementary Figure S3. Proportions of the classical neuroblastoma cell line, KCNR cells expressing GFP were detected using flow cytometry, 5-7 days following lentiviral vector transduction compared to wild type counterparts.

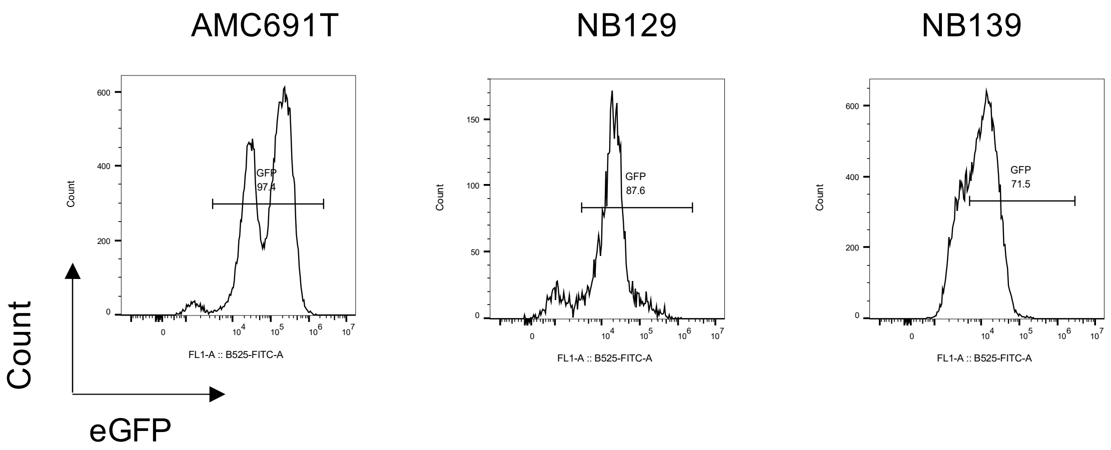

Supplementary Figure S4. Proportions of neuroblastoma organoids AMC691T , NB129 and NB139 expressing GFP were detected using flow cytometry.

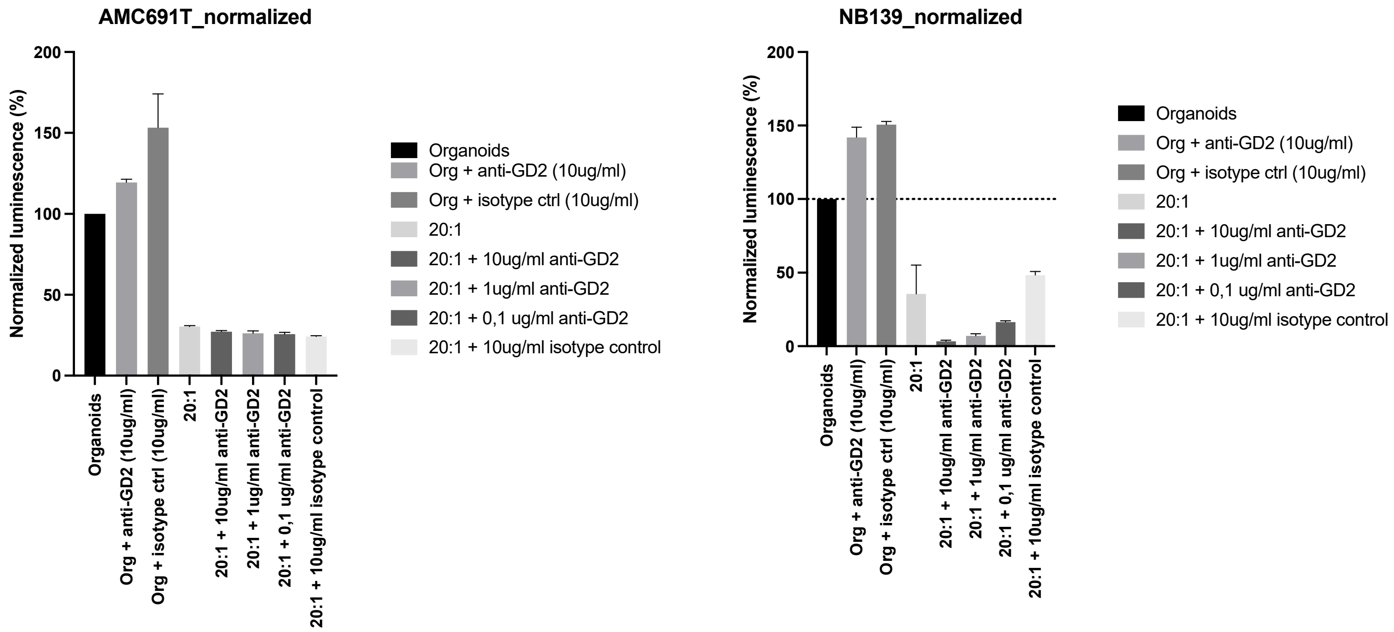

Supplementary Figure S5. Effect of Dinutuximab treatment on neuroblastoma cells cocultured with PBMCs. Chemiluminescence-based cell viability was measured using the ADCC luciferase assay. The viability of neuroblastoma cells were compared with cocultured neuroblastoma cells with PBMCs and in the presence dinutuximab, an FDA approved monoclonal antibody targeting GD2 antigen, as well as in the presence of isotype control. Tumour cells were exposed for 4 hours to 0.1, 1 and 10 ug/mL of dinutuximab in a GD2-expressing organoid line NB139 (right) and in a GD2-deficient organoid line AMC691T (left). All experiments were performed in triplicate. Error bars represent standard deviation values.

Supplementary videos

Supplementary video 1. coculture of GFP-ffLuc autologous NB tumour NB139 with peripheral blood T cells.

https://drive.google.com/file/d/1lGw-Wob6qIEU-8CFKvYYB_jzAogkSCDf/view?usp=sharing
